# Adenopathy and Extensive Skin Patch Overlying a Plasmacytoma (AESOP) Syndrome Associated With a Solitary Bone Plasmacytoma of the Left Scapula: A Rare Paraneoplastic Presentation Managed With High-Precision Radiotherapy

**DOI:** 10.7759/cureus.116234

**Published:** 2026-09-14

**Authors:** Aaditya Prakash, Amitabh Kumar Upadhyay, Chitra Sukumari, Pravesh Kumar, Subhankar Show, Ajithkumar M, Abhijin Satheeshkumar

**Affiliations:** 1 Radiation Oncology, Tata Main Hospital, Jamshedpur, IND; 2 Medical Oncology, Tata Main Hospital, Jamshedpur, IND; 3 Radiation Oncology, Meherbai Tata Memorial Hospital, Jamshedpur, IND

**Keywords:** aesop syndrome, ebrt, image-guided radiotherapy (igrt), imrt, organ-at-risk sparing, paraneoplastic syndrome, plasmacytoma, poems diagnosis, radiotherapy, rare cancers

## Abstract

Adenopathy and extensive skin patch overlying a plasmacytoma (AESOP) syndrome is a rare paraneoplastic manifestation characterized by three cardinal components: the underlying pathology (plasma cell dyscrasia), the cutaneous lesion (vascular cutaneous lesion), and the skeletal/nodal manifestation (solitary plasmacytoma/regional lymphadenopathy). Scapular involvement is exceptionally uncommon and may represent an early indicator of progression within the polyneuropathy, organomegaly, endocrinopathy, monoclonal plasma cell disorder, and skin changes (POEMS) spectrum.

A 47-year-old male with essential hypertension presented with a 12-month history of an insidiously expanding, progressive reddish-brown skin patch over the left anterior chest wall and upper lateral torso, together with localized pain in the left scapular region. After the patient’s history and examination, further workup for malignancy was advised. A baseline ¹⁸F-FDG PET/CT (fluorine-18 fluorodeoxyglucose positron emission tomography/computed tomography) scan demonstrated an avidly hypermetabolic, destructive osteolytic bone lesion strictly localized to the body of the left scapula (maximum standardized uptake value (SUV_max_) 18.3), alongside hypermetabolic left axillary lymphadenopathy (3.4 × 2.7 cm, SUV_max_ 3.5) and additional subcarinal, infraclavicular, and pretracheal hypermetabolic lymph nodes. A core biopsy of the left scapular mass confirmed a plasma cell neoplasm featuring dense sheets and cords of cells with plasmacytoid morphology. Immunohistochemistry (IHC) displayed absolute CD138 positivity and strict lambda light chain restriction while remaining negative for epithelial markers (AE1/AE3). Left axillary lymph node evaluation revealed reactive lymphoid cell proliferation with a preserved follicular dendritic network, successfully ruling out low-grade B-cell non-Hodgkin lymphoma. Bone marrow aspirate showed normocellular bone marrow with no evidence of lymphoma/myeloma (2-3% plasma cells). Laboratory evaluation confirmed a profound monoclonal gammopathy and severe light-chain distortion.

The patient was finally diagnosed with AESOP syndrome secondary to a solitary plasma cell neoplasm of the left scapula with minimal bone marrow involvement. He was successfully treated with a definitive dose of precision-based external beam radiotherapy using image-guided intensity-modulated radiotherapy (IMRT), targeting the left scapular lesion to a total curative dose of 50.4 Gy in 28 fractions and utilizing conformal beam arrangements to safeguard adjacent organs at risk (OARs), including the lung, heart, and spinal cord. Follow-up imaging confirmed complete structural, biochemical, and metabolic remission.

This case highlights the rare scapular manifestation of AESOP syndrome and emphasizes the importance of recognizing characteristic cutaneous findings as a clue to an underlying plasma cell neoplasm. Prompt diagnosis through clinicopathological correlation and histopathological confirmation, followed by definitive radiotherapy, resulted in durable local disease control and resolution of paraneoplastic manifestations.

## Introduction

In 2003, Lipsker et al. first formalized adenopathy and extensive skin patch overlying a plasmacytoma (AESOP) syndrome as a distinct, rare paraneoplastic entity [1]. The clinical hallmark of this syndrome is a slowly expanding vascular cutaneous plaque or patch that develops directly over or contiguous to a deep-seated monoclonal plasma cell tumor [2].

Due to the overproduction of proangiogenic and proinflammatory cytokines, predominantly vascular endothelial growth factor (VEGF), from the localized deep osseous tumor, these cytokines diffuse vertically through the fascial planes into the overlying dermis, causing intense localized endothelial proliferation, hypervascularity, and mucin deposition within the skin layers without direct physical infiltration of malignant plasma cells into the skin itself [3].

Most of the documented cases involving skeletal lesions have been reported in anterior chest wall structures such as the ribs or sternum, but primary involvement of the scapula appears uncommon, with only a few cases reported in the literature [4]. Recognizing this cutaneous-skeletal link is of paramount clinical importance, as around one-third of patients presenting with AESOP syndrome concurrently meet or eventually meet the diagnostic criteria for POEMS syndrome (polyneuropathy, organomegaly, endocrinopathy, M-protein, skin changes) [3,5]. Early identification allows for immediate local therapies, such as definitive radiotherapy, which achieves excellent local control, reverses paraneoplastic skin changes, and mitigates the risk of systemic progression [3,6].

We report a rare case of AESOP syndrome secondary to a solitary plasmacytoma involving the left scapula in a middle-aged male. The diagnosis was established through clinical presentation and examination, imaging, histopathological confirmation, immunophenotypic characterization, and biochemical assessment. The patient was successfully treated with a definitive dose of image-guided intensity-modulated radiotherapy, resulting in complete clinical, metabolic, and biochemical remission.

## Case presentation

A 47-year-old male with a recent history of essential hypertension, well controlled on amlodipine 5 mg once daily, presented with a 12-month history of progressive skin changes. The patient initially reported a slowly expanding, asymptomatic, reddish-brown discoloration over the left anterior aspect of his chest wall, for which he was evaluated by a dermatologist. However, due to aggravation of symptoms in the form of a deep, localized ache in the left shoulder blade region that had worsened with heavy lifting over the last few weeks, he was referred to the oncology department with suspicion of malignancy. He denied any associated constitutional signs such as fever, unexplained weight loss, night sweats, or neurological deficits.

Clinical examination revealed a large, well-demarcated, indurated, and hyperpigmented erythematous-violaceous cutaneous patch tracking across the left lateral chest wall and upper anterior torso (Figure 1). The skin surface was smooth, with no signs of ulceration, scaling, or itching.

**Figure 1:**
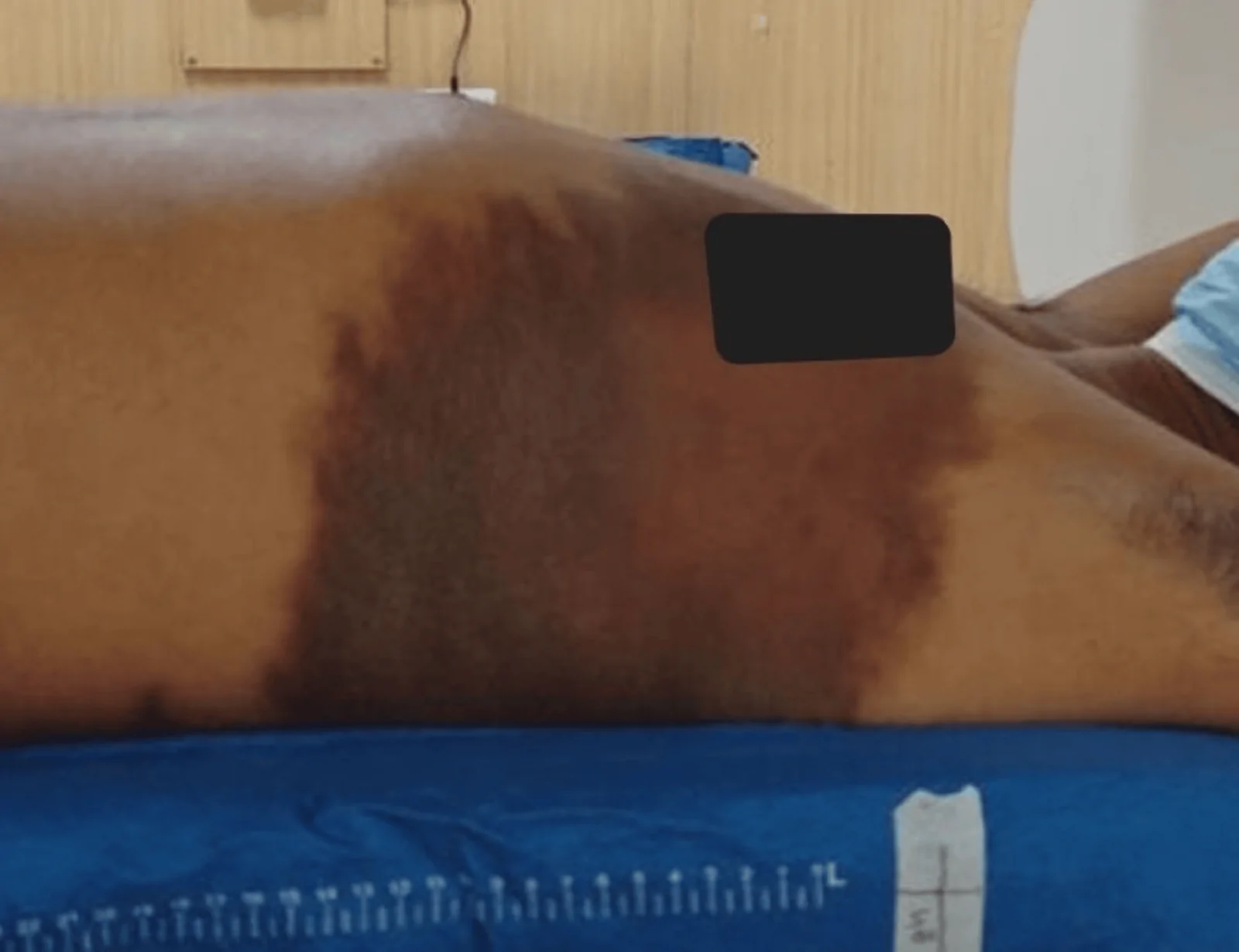
Pre-radiotherapy clinical photograph of the patient. A large, well-demarcated, indurated, and hyperpigmented erythematous-violaceous cutaneous patch is clearly visible across the left lateral chest wall and upper torso, extending contiguously toward the scapular region. This prominent cutaneous manifestation directly overlies the site of the deep-seated solitary plasmacytoma.

Palpation identified significant, firm, non-tender adenopathy in the left axilla. There was no lymphadenopathy outside the left axilla, and no systemic hepatosplenomegaly was noted. On further evaluation, a firm area beneath the left retroareolar region was noted, necessitating further diagnostic workup.

A left axillary lymph node (AXLN) biopsy initially raised suspicion for a low-grade B-cell non-Hodgkin lymphoma due to an atypical proliferation. However, a subsequent expert slide review performed at a referral laboratory favored reactive lymphadenitis (Figure 2). Immunohistochemistry (IHC) staining highlighted small lymphoid cell nodules positive for CD20, with parafollicular sheets of CD3-positive T cells. IgD and BCL2 were positive in sheets within the parafollicular areas, while CD10 and cyclin D1 were entirely negative. The MIB-1 (Ki-67) labeling index was 2-3%. A biopsy of the left breast lesion confirmed benign ductal units, and a biopsy of the anterior chest wall skin patch was negative for malignancy.

**Figure 2:**
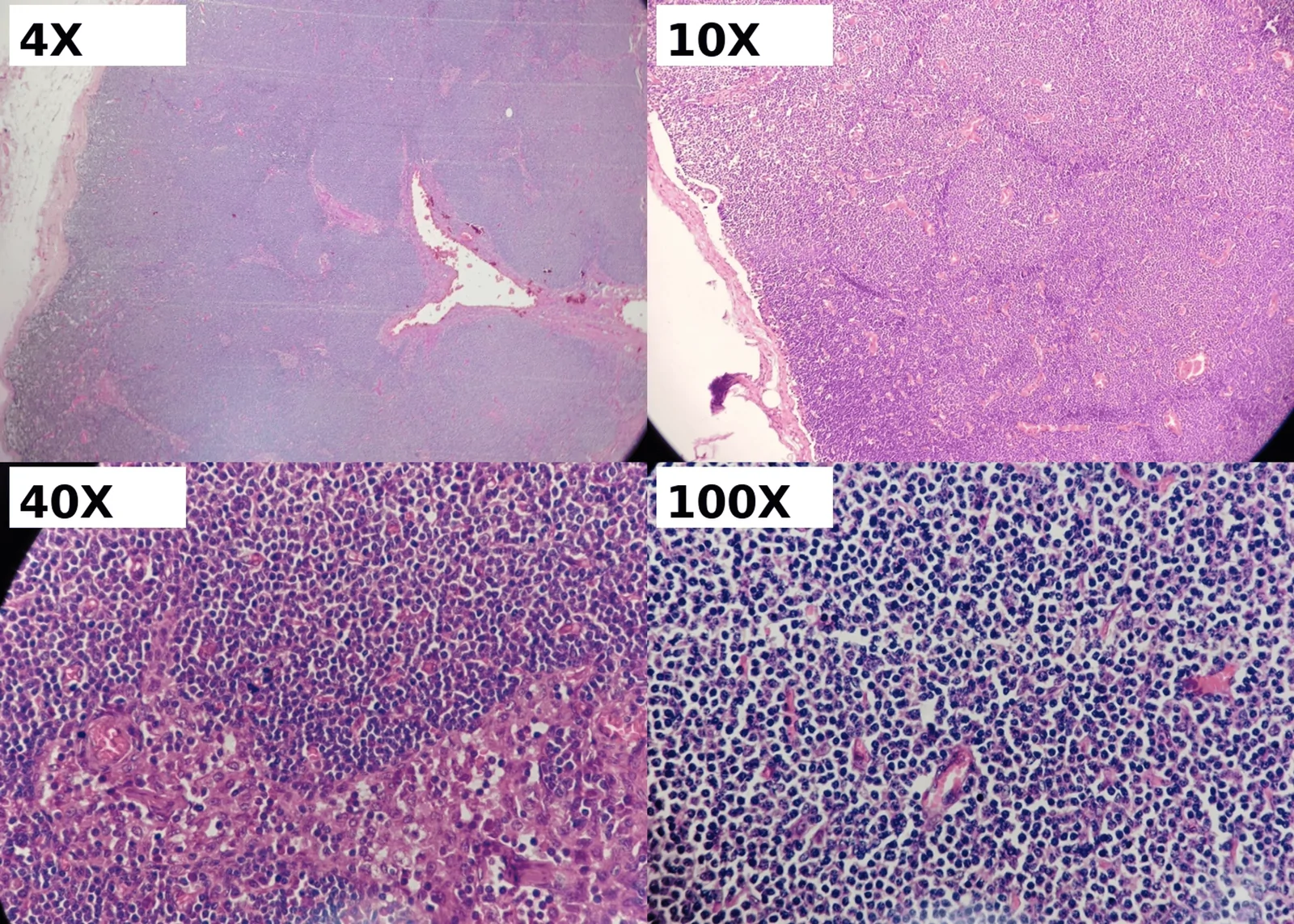
Hematoxylin and eosin (H&E) stained section of reactive lymphadenitis in 4x, 10x, 40x, and 100x power view.

To evaluate potential infectious etiologies, a bronchoalveolar lavage (BAL) was performed. BAL analysis revealed negative GeneXpert and fungal staining. The BAL galactomannan index was 0.66, which, while positive, did not meet the EORTC/MSGERC cutoff (≥1.0) for probable invasive aspergillosis. The results are shown in Table 1. 

**Table 1:** Microbiological work up of bronchoalveolar lavage sample.

Investigation	Result
GeneXpert	Negative
Fungal stains	Negative
Acid-fast bacilli (AFB) smear	Negative
afb culture	No growth
1,3-Beta-D-glucan	Negative
Galactomannan antigen	Positive (Index: 0.663)

A baseline ¹⁸F-FDG PET/CT scan demonstrated extensive metabolically active disease. The study revealed a highly aggressive lytic lesion involving the left scapula, showing intense hypermetabolic activity with a maximum standardized uptake value (SUV_max_) of 18.3 (Figure 3). In addition, a soft tissue lesion was identified in the left retroareolar region involving the lower outer quadrant of the breast, associated with mild skin thickening and measuring 2.2 × 1.1 cm, with relatively low metabolic activity (SUV_max_ 1.6). A low-grade left axillary lymph node measuring 3.4 × 2.7 cm with an SUV_max_ of 3.5 was also identified.

**Figure 3:**
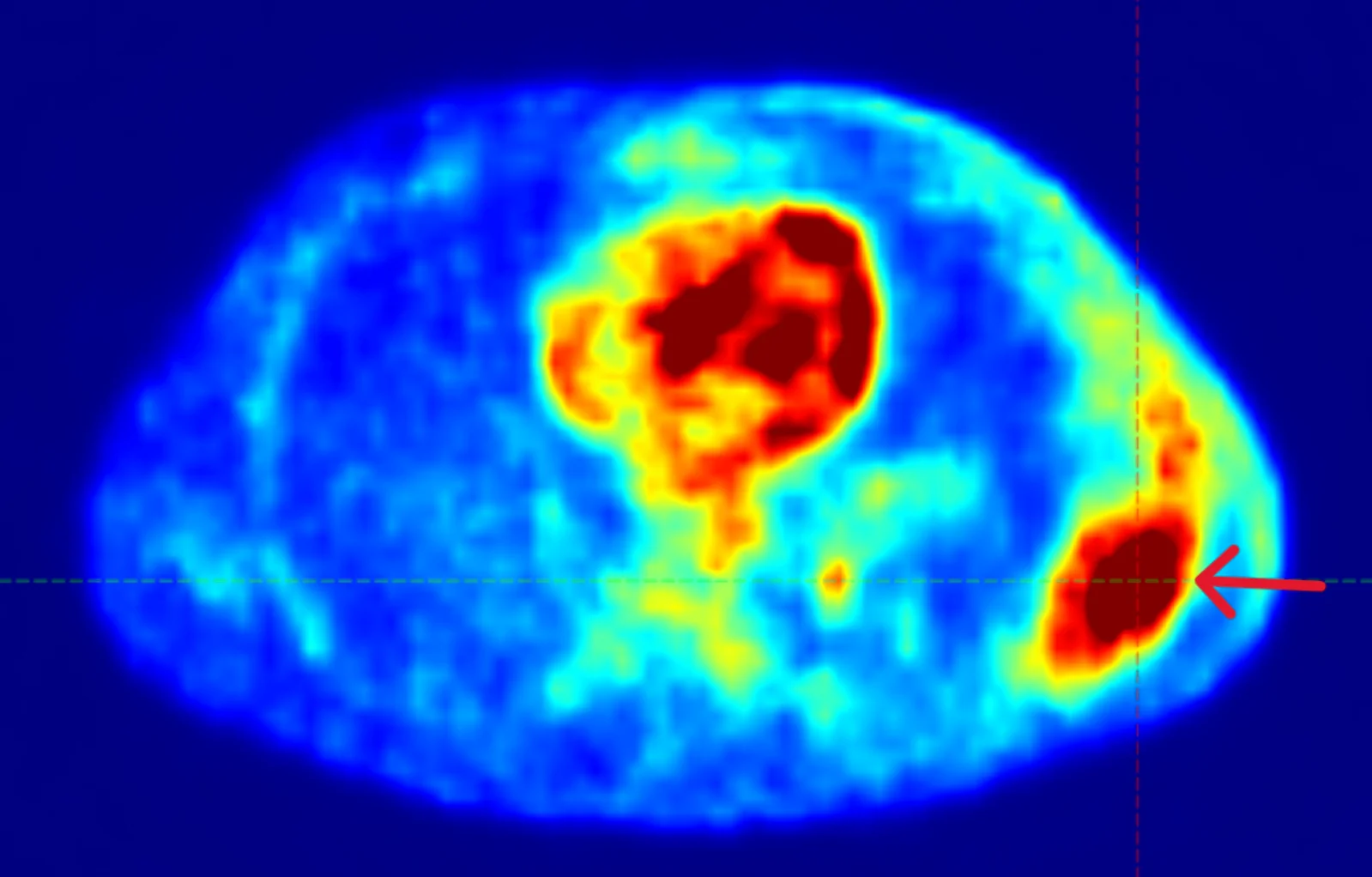
Baseline ¹⁸F-FDG PET/CT scan (axial view). Red arrow demonstrates intense, aggressive hypermetabolic tracer uptake (SUVmax 18.3) localized to the lytic lesion within the body of the left scapula, along with hypermetabolic regional thoracic lymph node involvement.

Endocrine workup was within normal physiological limits. However, serum protein electrophoresis (SPEP) and immunofixation revealed a profound monoclonal gammopathy and severe light chain distortion, as shown in Table 2 and Figure 4. 

**Figure 4:**
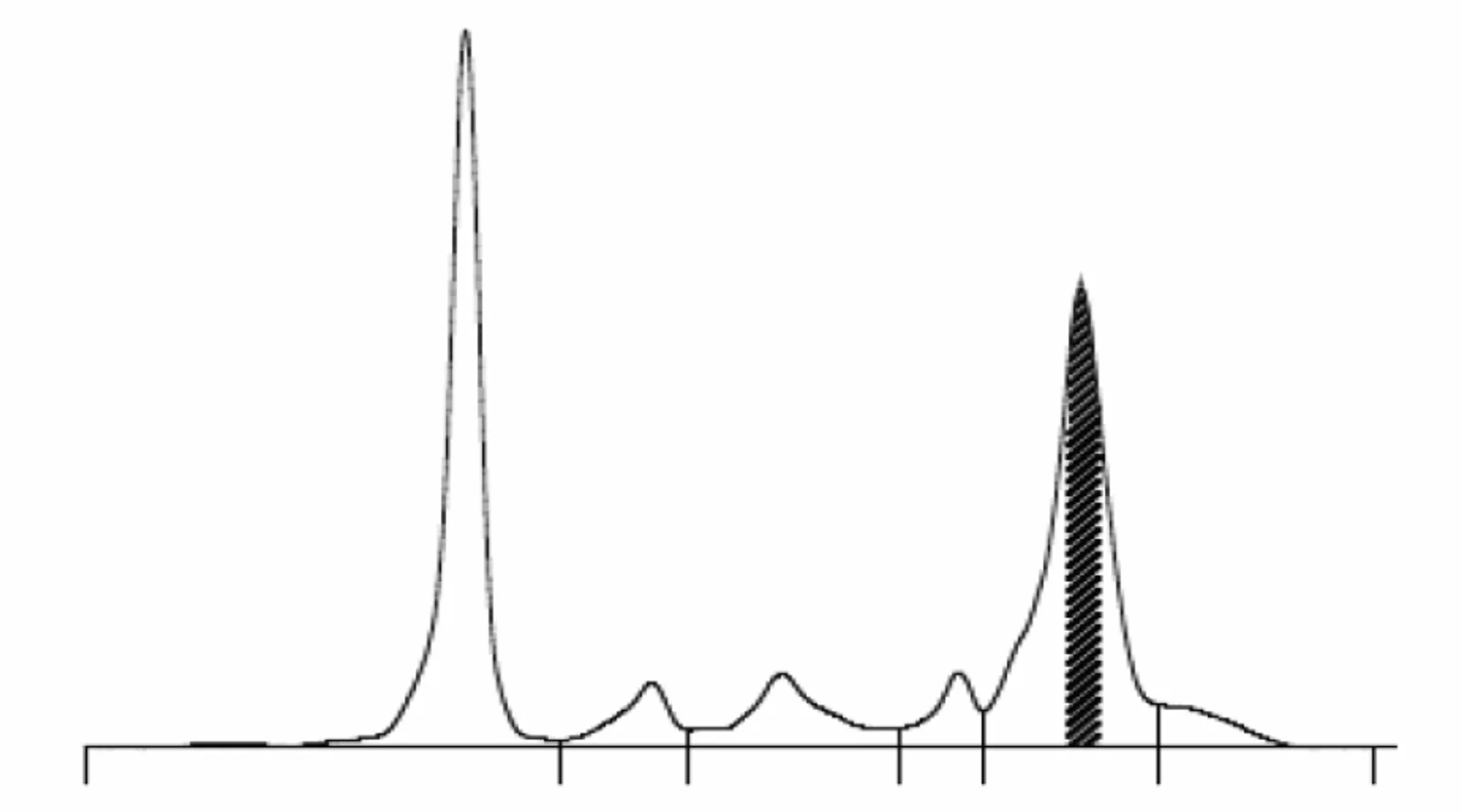
Pre-treatment Serum protein electrophoresis (SPEP). From left to right the peaks denotes, the albumin, α1-, α2-, β-, and γ-globulin fractions. The pre-treatment tracing demonstrates a prominent γ-region monoclonal peak (M-spike), corresponding to an IgG lambda paraprotein produced by the solitary plasmacytoma.

**Table 2:** Pretreatment serum protein electrophoresis (SPEP) report. *κ/λ ratio remained within the conventional reference range in this case, but the presence of a monoclonal M-protein together with histopathologically confirmed tissue biopsy supported the diagnosis of a clonal plasma-cell neoplasm.

Parameter	Value	Unit	Reference Range
Monoclonal M-band	500	mg/dL	0 mg/dL
Kappa (κ) light chains	819.00	mg/dL	170-370 mg/dL
Lambda (λ) light chains	1240.00	mg/dL	90-210 mg/dL
Free kappa (κ)	34.02	mg/L	3.3-19.4 mg/L
Free lambda (λ)	84.53	mg/L	5.7-26.3 mg/L
IgG	1620.0	mg/dL	700-1600 mg/dL
IgA	222	mg/dL	70-400 mg/dL
IgM	80.2	mg/dL	40-230 mg/dL
Kappa/lambda (κ/λ) ratio*	0.40	Ratio	0.26-1.65

A core biopsy of the left scapula demonstrated a plasma cell neoplasm. Microscopic examination revealed a dense tumor growing in diffuse sheets and cords with classical plasmacytoid morphology (Figure 5). On immunohistochemistry performed at a referral laboratory, the cells were strongly positive for CD138 and demonstrated strict lambda (λ) light-chain restriction while being entirely negative for epithelial markers (AE1/AE3). Bone marrow aspirate showed normocellular bone marrow with no evidence of lymphoma/myeloma (2-3% plasma cells).

**Figure 5:**
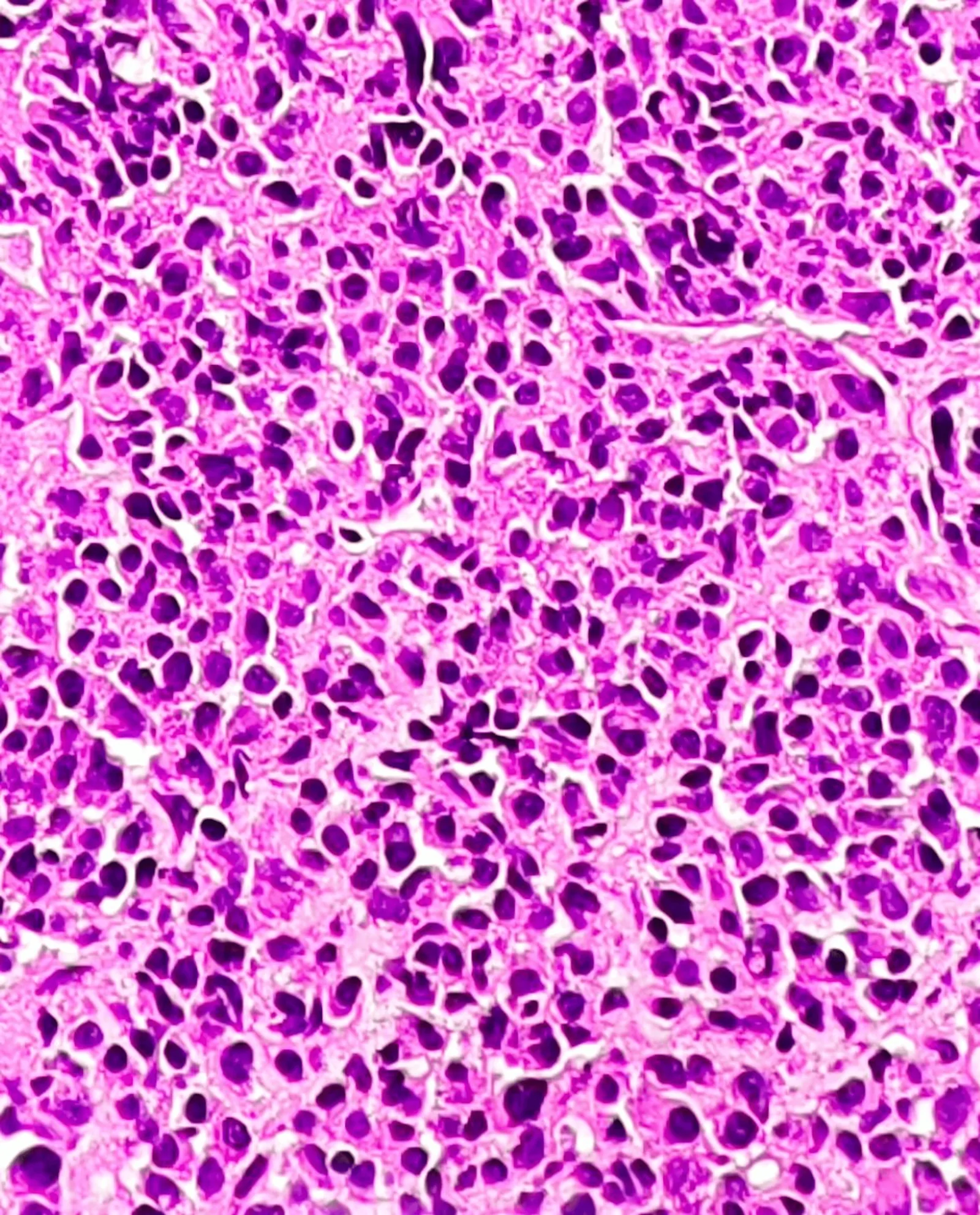
Hematoxylin and eosin (H&E) stained section of left scapular lesion 100x power view suggesting plasmacytoma.

Diagnosis and management

The triad of an extensive, hyperpigmented vascular skin patch, regional adenopathy, and an underlying solitary bone plasmacytoma confirmed the diagnosis of AESOP syndrome secondary to a primary plasma cell neoplasm of the left scapula with minimal bone marrow involvement.

The patient was planned for definitive-dose IMRT with a planning target volume (PTV) dose of 50.4 Gy, intended to be delivered in 28 fractions at 1.8 Gy per fraction over a 5.5-week period.

The treatment plan was designed on an advanced computerized treatment planning system (TPS) utilizing an inverse-planned, multi-field conformal beam arrangement (Figures 6, 7). For better coverage of the target volume during respiratory movement, a bolus was added over the chest wall. Multi-angle dynamic coplanar fields were optimized using a TrueBeam linear accelerator platform with 6 MV photon energy. Treatment planning provided optimal PTV coverage with the 95% isodose volume and effective sparing of surrounding critical structures through rapid dose fall-off. Dosimetric parameters included a spinal cord D_max_ of 35 Gy, left lung D_mean_ of 20 Gy, heart D_mean_ of 8.8 Gy, and heart D_max_ of 14.7 Gy.

**Figure 6:**
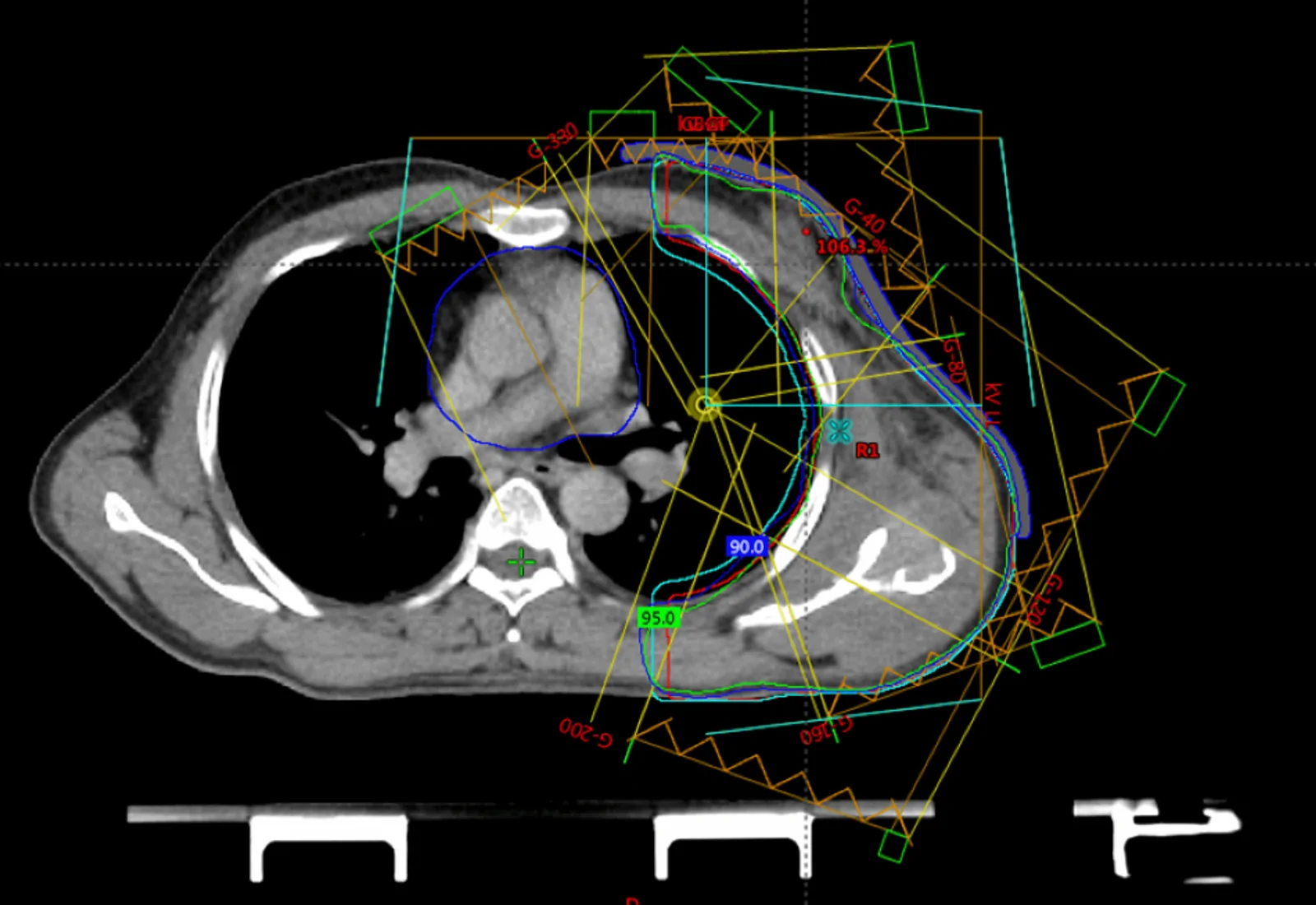
Axial view of the 3D conformal/intensity-modulated radiotherapy (IMRT) treatment plan on the Eclipse Treatment Planning System (TPS). The image demonstrates the optimized beam arrangement using multi-angle coplanar fields designed to deliver a highly uniform dose distribution to the planning target volume (PTV) of the left scapular and adjacent chest wall region. The color-coded isodose lines show strict dose conformity to the primary lesion while successfully sparing critical adjacent organs at risk (OARs), including the left lung parenchyma, heart, and spinal cord.

**Figure 7:**
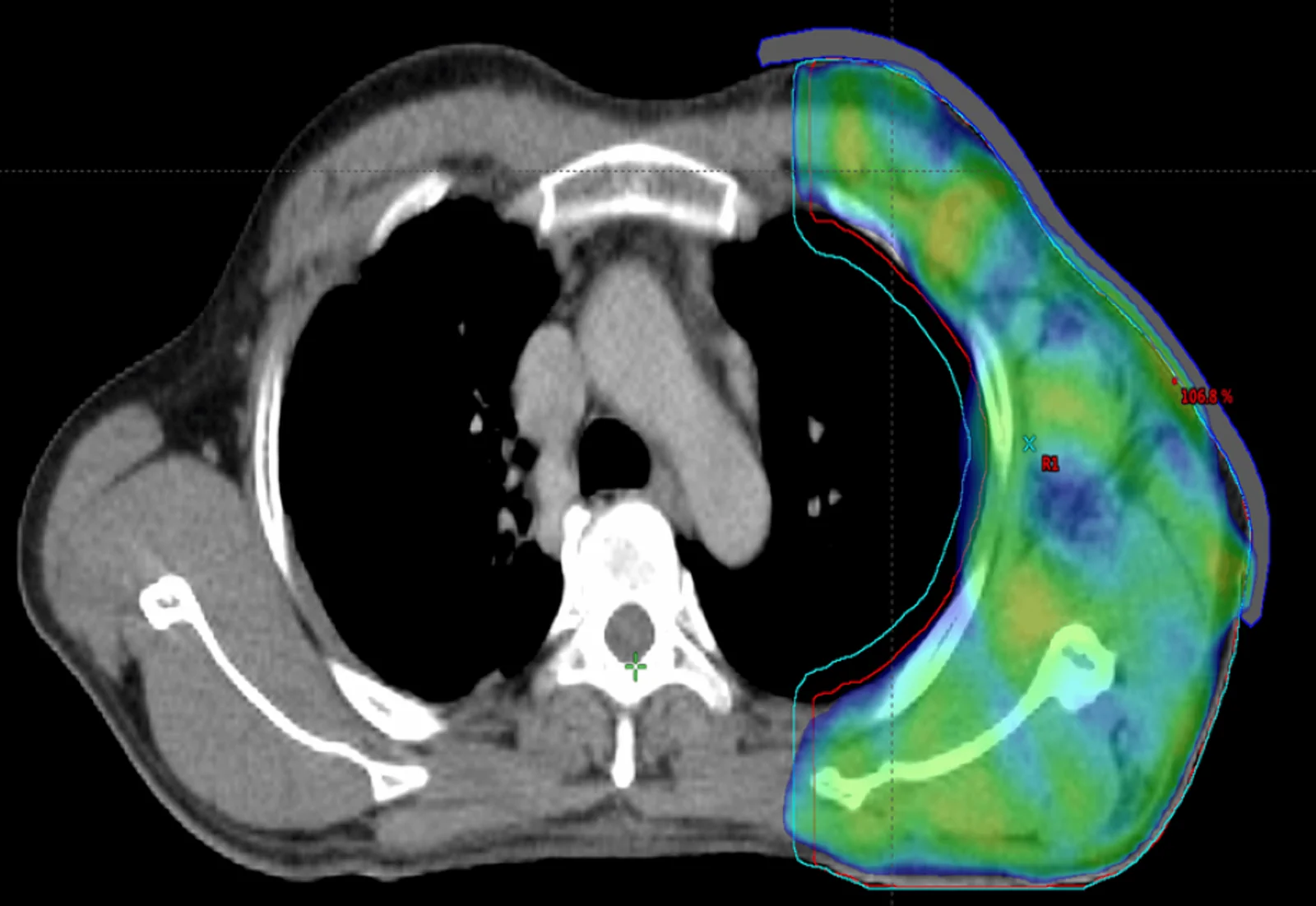
Axial slice of the 3D conformal/intensity-modulated radiotherapy (IMRT) plan from the Eclipse Treatment Planning System (TPS). The plan details the integration of a custom superficial bolus (highlighted by the purple/cyan boundary contours) applied directly over the left anterior chest wall and mammary region. This bolus modifies beam characteristics to eliminate the skin-sparing effect of high-energy photons, ensuring that the 95% to 100% target isodose clouds adequately cover the extensive cutaneous manifestations of AESOP syndrome while protecting deep-seated pulmonary structures.

Clinical outcome and follow-up

At the three-month post-treatment follow-up, the patient reported complete resolution of his left shoulder pain during activity. Clinical inspection showed that the large erythematous-violaceous skin patch across the left anterior chest wall and lateral torso had entirely faded, leaving normal, healthy skin texture with only minor residual post-inflammatory hyperpigmentation (Figure 8). 

**Figure 8:**
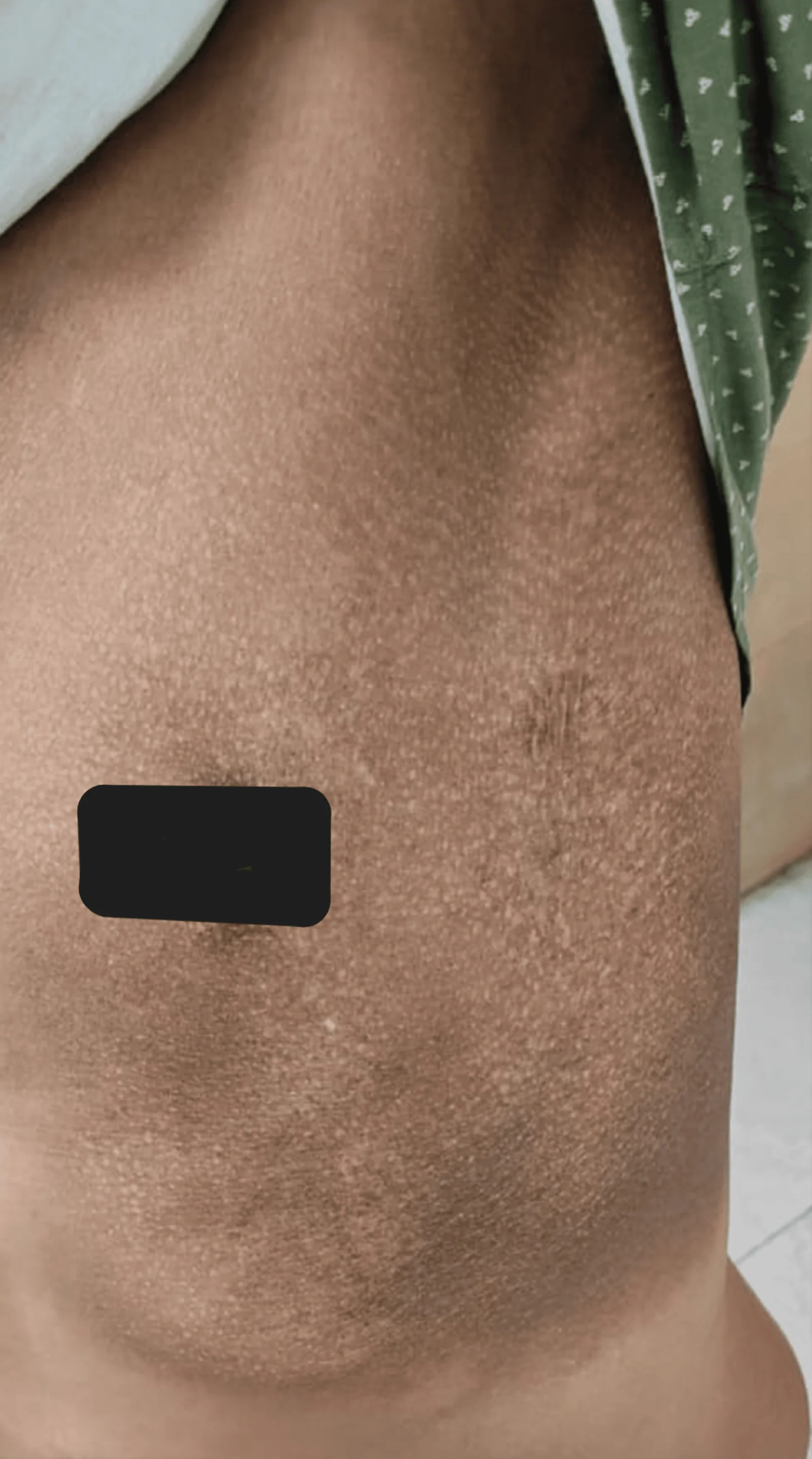
Post-radiotherapy clinical photograph of the patient at three-month follow-up. Examination reveals substantial clinical improvement, characterized by near-complete resolution of the large, indurated erythematous-violaceous patch across the left lateral chest wall and upper torso. The skin shows normal texture with residual, fading post-inflammatory hyperpigmentation.

Follow-up serum and biomedical markers revealed complete normalization and the total disappearance of the IgG lambda M-protein band (Figure 9).

**Figure 9:**
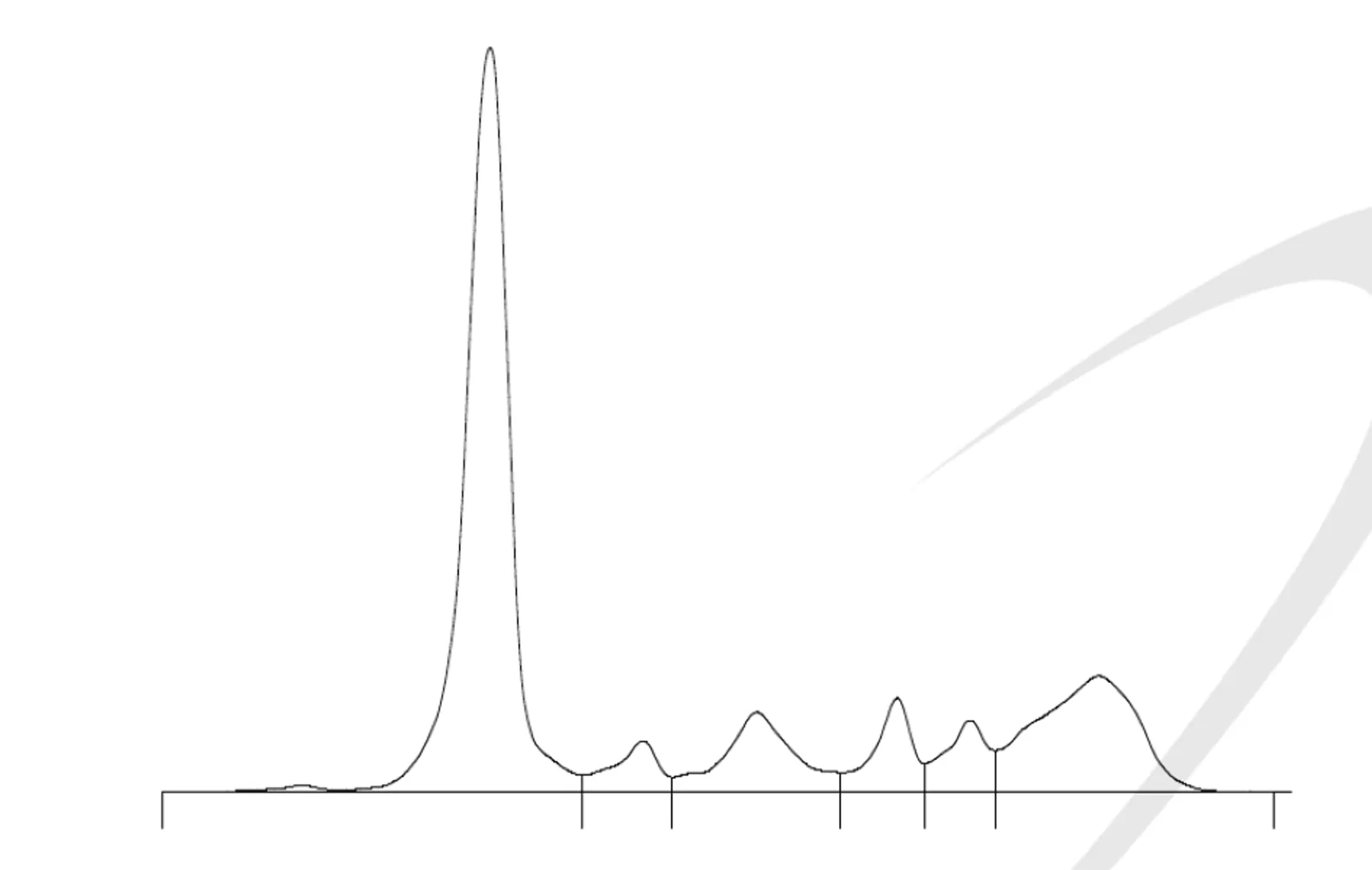
Post-treatment Serum protein electrophoresis (SPEP). From left to right the peaks denotes, the albumin, α1-, α2-, β-, and γ-globulin fractions. Post-treatment SPEP shows complete disappearance of the M-spike with restoration of a normal polyclonal γ-globulin profile, consistent with complete biochemical response.

A post-radiation follow-up ¹⁸F-FDG PET/CT scan performed after three months showed a remarkable therapeutic response, demonstrating structural stabilization and a dramatic reduction in hypermetabolic tracer uptake within the left scapular lesion and regional lymph node chains (Figure 10).

**Figure 10:**
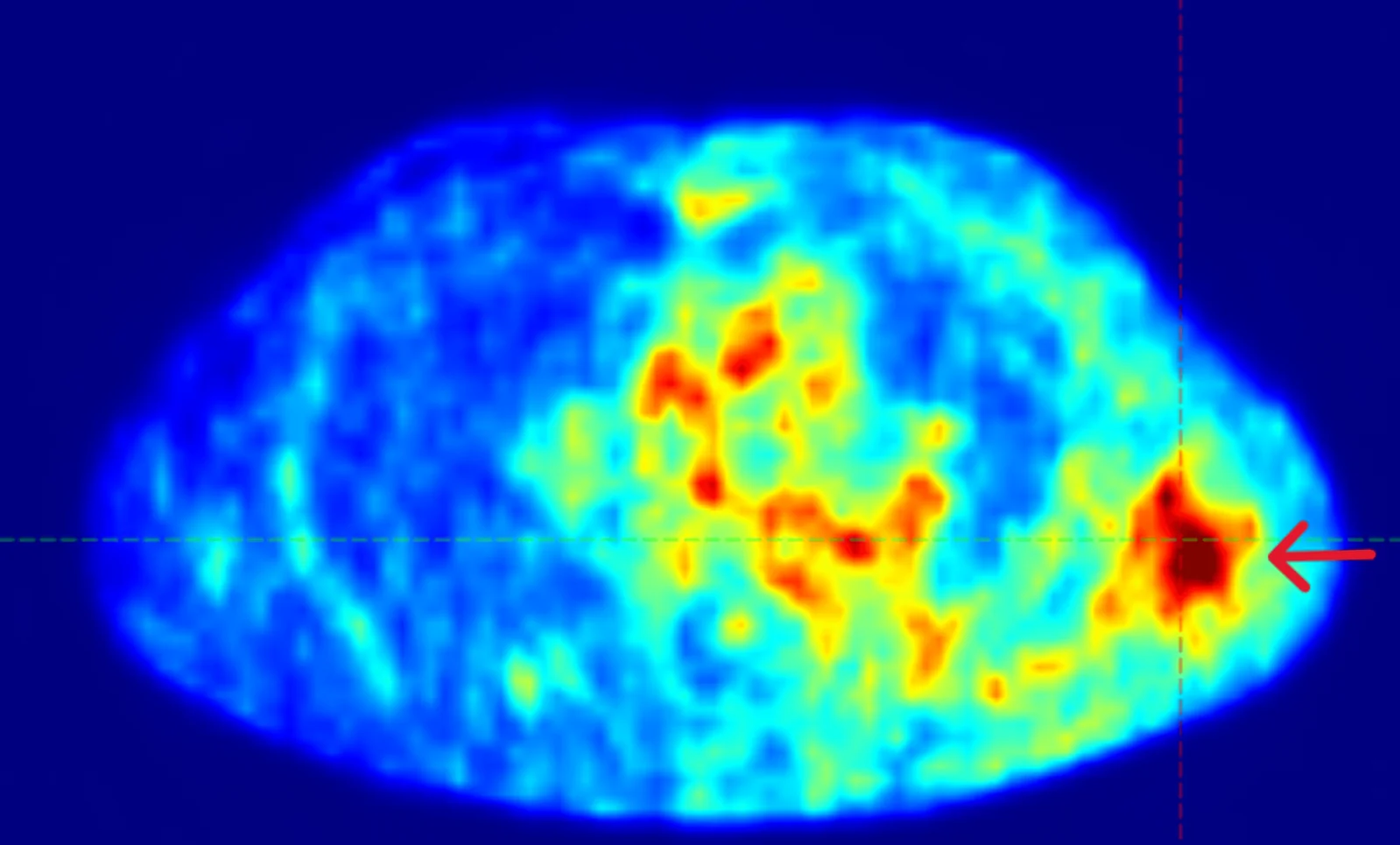
Post-radiation staging ¹⁸F-FDG PET/CT scan (axial view) at three months post-treatment. Red arrow shows early therapeutic response, demonstrating significant regression and a prominent decline in metabolic intensity within the left scapular bone lesion and regional thoracic lymph node stations compared to pre-treatment levels.

The patient was maintained under close clinical and imaging surveillance. Long-term follow-up imaging performed approximately 15 months post-radiotherapy confirmed an enduring complete metabolic remission with complete resolution of any abnormal FDG avidity within the left scapular site (Figure 11). The patient remains in clinical, biochemical, and imaging remission without signs of progression to systemic multiple myeloma or POEMS syndrome. 

**Figure 11:**
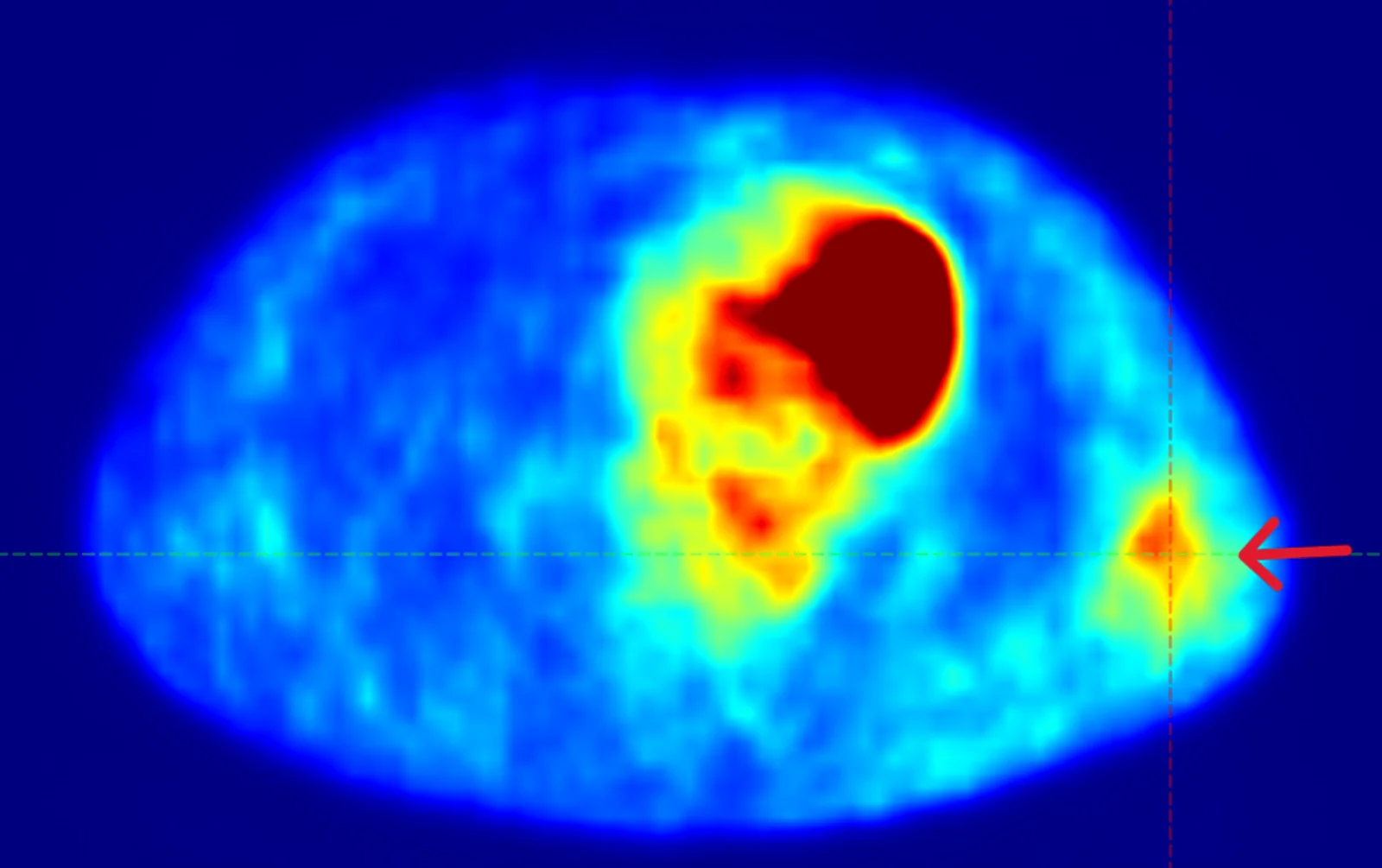
Long-term follow-up ¹⁸F-FDG PET/CT scan (axial view) at approximately 15 months post-treatment. Red arrow shows complete metabolic response with non FDG avidity at treated site.

## Discussion

This case illustrates an uncommon manifestation of a localized plasma cell neoplasm in which cutaneous and nodal abnormalities served as the initial indicators of an otherwise occult malignancy. The combination of a progressive vascular cutaneous lesion, regional lymphadenopathy, monoclonal gammopathy, and a metabolically active scapular lesion ultimately led to the diagnosis of a solitary plasmacytoma associated with AESOP syndrome. The unusual scapular location and favorable response to definitive radiotherapy emphasize the importance of recognizing this distinctive clinicopathological association at an early stage of disease evolution [3,7].

From an oncologic perspective, the cutaneous lesion can be viewed as a visible biological marker of an underlying plasma cell neoplasm arising from overproduction of proinflammatory cytokines such as VEGF. The absence of malignant plasma cells in skin biopsies and the resolution of cutaneous abnormalities following treatment of the plasmacytoma strongly support this cytokine-driven pathophysiological mechanism [6,7].

The scapula represents an uncommon site for solitary plasmacytoma. Most solitary bone plasmacytomas arise within marrow-rich axial skeletal structures such as the vertebral column, ribs, sternum, and pelvis. Consequently, scapular involvement may delay recognition, particularly when dermatological manifestations overshadow skeletal symptoms during the initial clinical presentation. In the present case, the conspicuous cutaneous lesion prompted detailed multidisciplinary evaluation, ultimately leading to identification of a highly FDG-avid osteolytic lesion within the body of the left scapula.

Another clinically significant aspect of this case was the presence of regional and mediastinal hypermetabolic lymphadenopathy. Such findings may raise concern for lymphoma, metastatic disease, or coexistence of a secondary hematological malignancy. However, comprehensive histopathological and IHC analysis demonstrated preserved nodal architecture with reactive lymphoid hyperplasia and no evidence of a clonal lymphoid proliferation. This observation underscores the necessity of tissue confirmation before attributing lymph node enlargement to disseminated malignancy, particularly in rare plasma-cell-associated syndromes where reactive adenopathy may constitute part of the disease spectrum [1,7,8].

Several investigators have proposed that AESOP syndrome may represent an early or incomplete manifestation within the broader POEMS spectrum. Therefore, patients presenting with localized cytokine-mediated skin changes, monoclonal protein production, or unexplained lymphadenopathy should undergo comprehensive evaluation for systemic disease. Early recognition of these precursor manifestations may allow therapeutic intervention before the onset of irreversible neurological, endocrine, or visceral complications associated with advanced POEMS syndrome [5,7].

From a radiation oncology perspective, the present case highlights the remarkable radiosensitivity of solitary plasmacytoma. When diagnosed before progression to overt multiple myeloma, localized plasma cell neoplasms can often be effectively managed with definitive radiotherapy alone. Delivery of 50.4 Gy in 28 fractions in this case achieved excellent local control while maintaining acceptable dose constraints to surrounding critical organs, including the heart, lungs, and spinal cord. The use of conformal image-guided treatment planning further optimized target coverage and minimized treatment-related toxicity. These outcomes are consistent with published literature demonstrating durable local control rates exceeding 85%-90% following appropriately delivered definitive radiotherapy [5,7].

The dramatic clinical response observed following treatment further supports the concept that the cutaneous and nodal manifestations were directly linked to the underlying plasma cell neoplasm. Resolution of the vascular skin patch, disappearance of the monoclonal protein, normalization of laboratory parameters, and complete metabolic remission on follow-up PET/CT collectively indicated successful eradication of the disease-driving clonal plasma cell population. Long-term imaging confirmation of sustained remission further emphasizes the effectiveness of early definitive local therapy in appropriately selected patients.

Overall, this case highlights some important oncologic principles. First, unusual cutaneous findings may serve as the earliest clinical manifestation of an otherwise occult plasma cell malignancy. Second, AESOP syndrome should prompt systematic investigation for associated plasma cell dyscrasia and potential progression toward POEMS syndrome [7-9].

## Conclusions

In conclusion, AESOP syndrome associated with a solitary plasmacytoma of the left scapula constitutes a rare but pathognomonic oncologic presentation of localized plasma cell neoplasia. The coexistence of a characteristic overlying vascular cutaneous lesion and regional lymphadenopathy should prompt immediate investigation for an underlying plasma cell neoplasm and thorough assessment for POEMS syndrome. Recognition of this uncommon clinicopathological entity facilitates early diagnosis, accurate staging, and timely institution of definitive radiotherapy, which offers a high likelihood of durable local control, resolution of paraneoplastic manifestations, and prevention of progression to systemic disease.
